# Supplementary material for: Generating high quality libraries for DIA MS with empirically corrected peptide predictions
Source: Nat Commun. 2020 Mar 25;11:1548. doi: 10.1038/s41467-020-15346-1 (PMC7096433; doi:10.1038/s41467-020-15346-1)
Supplement: Supplementary file 3 — Description of Additional Supplementary Files [file 41467_2020_15346_MOESM3_ESM.docx]

**Description of Additional Supplementary Files**

**File Name: Supplementary Data 1**

**Description:** HeLa-specific missense variants detected in gas-phase fractionated. DIA data.

**File Name: Supplementary Data 2**

**Description:** Repeatedly measured P. falciparum proteins with single-injection DIA.Previous observations of proteins in the PlasmoDB compendium from different studies at various asexual, sexual, and mosquito stages are annotated.

**File Name: Supplementary Data 3**

**Description:** Descriptions of each MS/MS raw file, including acquisition type, biological sample, and replicate type/number.
