# Supplementary material for: Generating high quality libraries for DIA MS with empirically corrected peptide predictions
Source: Nat Commun. 2020 Mar 25;11:1548. doi: 10.1038/s41467-020-15346-1 (PMC7096433; doi:10.1038/s41467-020-15346-1)
Supplement: Supplementary file 7 — Reporting Summary [file 41467_2020_15346_MOESM7_ESM.pdf]

natureresearch

Corresponding author(s): Brian C. Searle

Last updated by author(s): Februrary 26, 2020

Reporting Summary

Nature Research wishes to improve the reproducibility of the work that we publish. This form provides structure for consistency and transparency in reporting. For further information on Nature Research policies, see Authors & Referees and the Editorial Policy Checklist .

Statistics

For all statistical analyses, confirm that the following items are present in the figure legend, table legend, main text, or Methods section.

n/a	Confirmed	
☐	☒	The exact sample size (*n*) for each experimental group/condition, given as a discrete number and unit of measurement
☐	☒	A statement on whether measurements were taken from distinct samples or whether the same sample was measured repeatedly
☒	☐	The statistical test(s) used AND whether they are one- or two-sided<br>*Only common tests should be described solely by name; describe more complex techniques in the Methods section.*
☒	☐	A description of all covariates tested
☒	☐	A description of any assumptions or corrections, such as tests of normality and adjustment for multiple comparisons
☐	☒	A full description of the statistical parameters including central tendency (e.g. means) or other basic estimates (e.g. regression coefficient) AND variation (e.g. standard deviation) or associated estimates of uncertainty (e.g. confidence intervals)
☒	☐	For null hypothesis testing, the test statistic (e.g. *F*, *t*, *r*) with confidence intervals, effect sizes, degrees of freedom and *P* value noted<br>*Give P values as exact values whenever suitable.*
☒	☐	For Bayesian analysis, information on the choice of priors and Markov chain Monte Carlo settings
☒	☐	For hierarchical and complex designs, identification of the appropriate level for tests and full reporting of outcomes
☒	☐	Estimates of effect sizes (e.g. Cohen's *d*, Pearson's *r*), indicating how they were calculated

Our web collection on statistics for biologists contains articles on many of the points above.

Software and code

Policy information about availability of computer code

Data collection	Thermo XCalibur 2.9.0.2926 (malaria) and XCalibur 3.0.2041 (yeast)
Data analysis	ProteoWizard version 3.0.18299, Trans Proteome Pipeline 5.1.0, Comet version 2015.02v2, MaxQuant 1.6.3.4, Skyline version 3.1.0.7382, EncyclopeDIA (https://bitbucket.org/searleb/encyclopedia), and Prosit (https://github.com/kusterlab/prosit)

For manuscripts utilizing custom algorithms or software that are central to the research but not yet described in published literature, software must be made available to editors/reviewers. We strongly encourage code deposition in a community repository (e.g. GitHub). See the Nature Research guidelines for submitting code & software for further information.

Data

Policy information about availability of data

All manuscripts must include a data availability statement. This statement should provide the following information, where applicable:

- Accession codes, unique identifiers, or web links for publicly available datasets
- A list of figures that have associated raw data
- A description of any restrictions on data availability

The raw data from the yeast and P. falciparum studies are available at MassIVE (MSV000084000) and file descriptions are listed in Supp. Data 3. The raw data from the HeLa reanalysis are available as originally published at MassIVE (MSV000082805).

Field-specific reporting

Please select the one below that is the best fit for your research. If you are not sure, read the appropriate sections before making your selection.

Life sciences study design

All studies must disclose on these points even when the disclosure is negative.

Sample size	The focus of this study was on technical variability of a data acquisition and analysis method, rather than biological variability. As such, technical replicates were acquired, rather than biological replicates.
Data exclusions	One sample was excluded from the malaria experiment due to poor enrichment. This is discussed in depth in Supplementary Figure 8.
Replication	The yeast experiment was a study of technical variability, where N=4 technical replicates were acquired from the same biological sample. The malaria experiment was a quantitative study of technical variability in a pool of N=3 biological replicates. All three samples were analyzed with DDA individually to build the DDA library.
Randomization	Data acquisition for the dilution curve was acquired in reverse order (smallest concentration first) to minimize carry over. Data acquisition of the replicates was performed in a DIA/DDA alternating fashion
Blinding	Blinding was not relevant to this study because there was no analysis of biological variation.

Reporting for specific materials, systems and methods

We require information from authors about some types of materials, experimental systems and methods used in many studies. Here, indicate whether each material, system or method listed is relevant to your study. If you are not sure if a list item applies to your research, read the appropriate section before selecting a response.

Materials & experimental systems

n/a	Involved in the study	
☒	☐	Antibodies
☐	☒	Eukaryotic cell lines
☒	☐	Palaeontology
☒	☐	Animals and other organisms
☒	☐	Human research participants
☒	☐	Clinical data

Methods

n/a	Involved in the study	
☒	☐	ChIP-seq
☒	☐	Flow cytometry
☒	☐	MRI-based neuroimaging

Eukaryotic cell lines

Policy information about cell lines

Cell line source(s)	Plasmodium falciparum strain NF54 cells were acquired from the S. Kappe lab at Seattle Children's Research Institute (originally from ATCC). Human O+ erythrocytes (RBCs) were obtained from Valley Biomedical (Winchester, VA; catalog number HP1002O).
Authentication	Cell lines were originally authenticated using genotyping.
Mycoplasma contamination	Cell lines were tested for Mycoplasma. Cells are also visually confirmed for the absence of bacteria by Giemsa stain.
Commonly misidentified lines (See ICLAC register)	none were used
